# Supplementary material for: Protopine-Type Alkaloids Alleviate Lipopolysaccharide-Induced Intestinal Inflammation and Modulate the Gut Microbiota in Mice
Source: Animals (Basel). 2024 Aug 5;14(15):2273. doi: 10.3390/ani14152273 (PMC11311078; doi:10.3390/ani14152273)
Supplement: Supplementary file 1 [file animals-14-02273-s001.zip › animals-3086183-supplementary.pdf]

Supplementary Files

Protopine total alkaloid alleviates lipopolysaccharide-induced intestinal inflammation and modulates the gut microbiota in mice

Jialu Huang[1,2], Meishan Yue[1,2], Yang Yang[2], Yisong Liu[2], Jianguo Zeng[1,2*]

[1]College of Veterinary Medicine, Shanxi Agricultural University, Jinzhong 030801, China;

[2]College of Veterinary Medicine, Hunan Agricultural University, Changsha 410128, China;

* Correspondence: Jianguo Zeng

E-mail: zengjianguo@hunau.edu.cn.

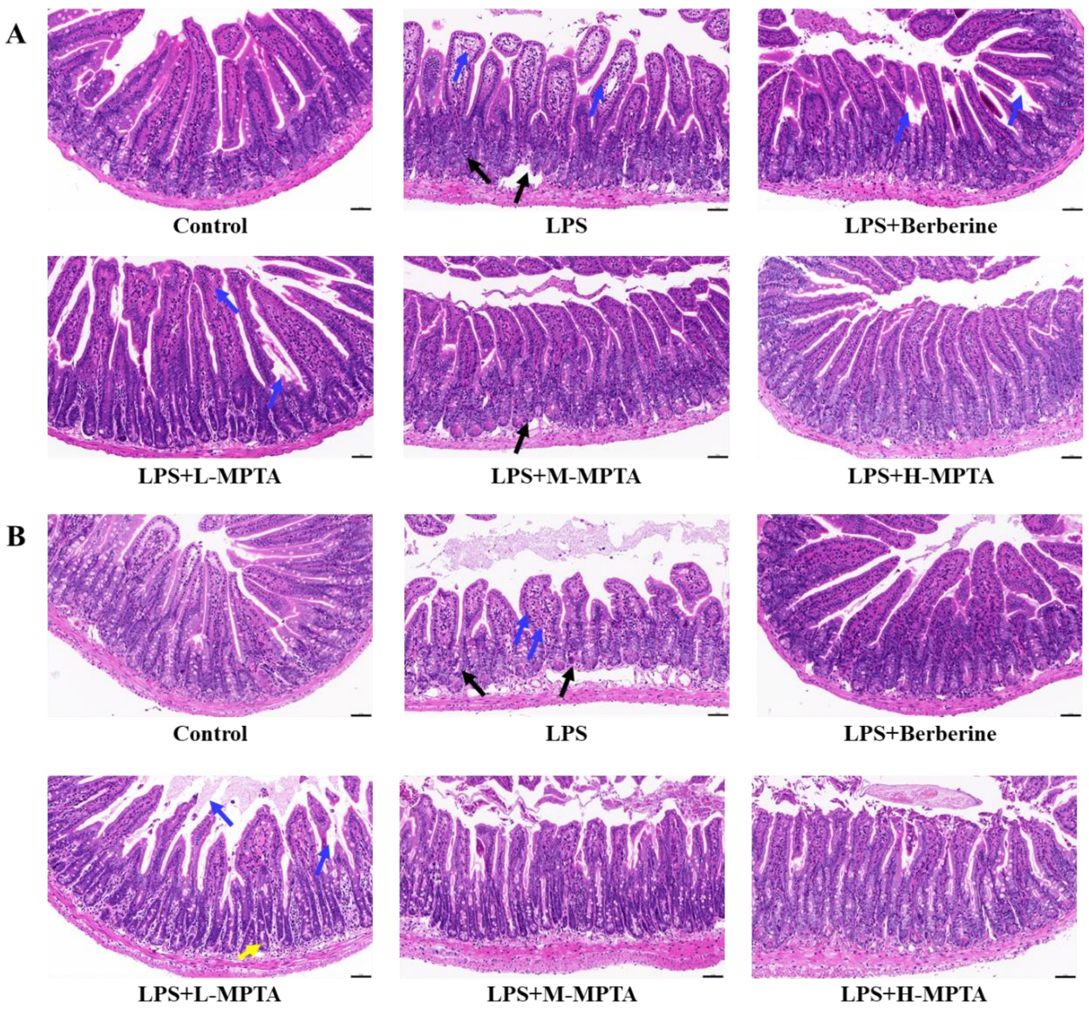

Figure S1. Effects of MPTA on the morphology of jejunum and ileum in LPS-induced mice. (A) Jejunal morphology with H&E staining, scale bar: 50 μm. (B) Ileal morphology with H&E staining, scale bar: 50 μm.

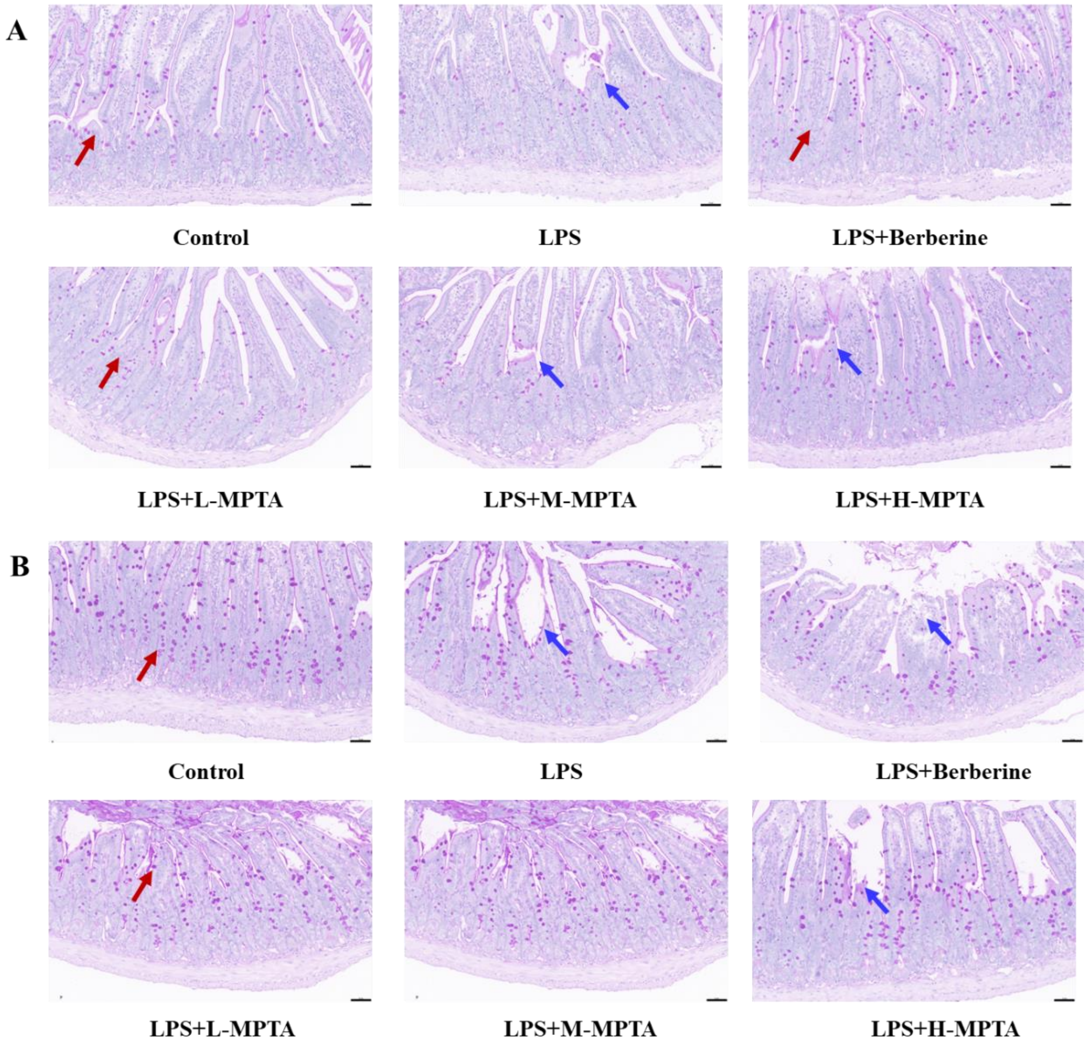

Figure S2. Effect of MPTA on GCs of jejunum and ileum in LPS-induced mice. (A) Jejunal goblet cells (GCs) with PAS staining, scale bar: 50 μm. (B) Ileal GCs with PAS staining, scale bar: 50 μm.

Table S1 The ratio of Firmicutes/ Bacteroidetes (F/B)

	Control	LPS	LPS+Berberine	LPS+L-MPTA	LPS+M-MPTA	LPS+H-MPTA
F/B	0.404	1.257	2.109	3.211	0.739	0.408

Table S2 Contents of different groups of short-chain fatty acids (mmol/L)

	Control	LPS	Berberine	LPS+L-MPTA	LPS+M-MPTA	LPS+H-MPTA
Acetic acid	4.4419±0.31328	2.4568±0.41829##	3.1595±0.48396**	2.7140±0.32191	2.9584±0.50254*	3.8481±1.52909**
Propionic acid	0.9220±0.17895	0.5581±0.10431##	0.7719±0.13972**	0.6253±0.12310	0.4788±0.06898	0.5283±0.14862
Isobutyric acid	0.0708±0.01646	0.0747±0.02160	0.1126±0.01569**	0.0849±0.02576	0.0592±0.01346	0.0717±0.02492
Butyric acid	0.5300±0.09911	0.4479±0.09359	0.6429±0.07372**	0.4505±0.06978	0.4144±0.17517	0.4275±0.17896
Isovaleric acid	0.0760±0.00814	0.1055±0.03820##	0.1393±0.01265**	0.1296±0.01772*	0.1257±0.02348	0.1156±0.02777
Valeric acid	0.0363±0.00374	0.0421±0.01835	0.0743±0.01221**	0.0510±0.01115	0.0324±0.01144	0.0366±0.01078
Volatile fatty acid	6.0767±0.30254	3.6850±0.66813##	4.9004±0.64987**	4.0556±0.29188	4.0630±0.54108	5.0279±1.88386**

Results are presented as mean values ± SEM (standard error of the mean).

The p-value < 0.01 (##) vs the control group, p-value < 0.05(*) or 0.01 (**) vs the LPS group.
